# Capturing Dynamic Assembly of Nanoscale Proteins During Network Formation

**DOI:** 10.1002/smll.202407090

**Published:** 2024-11-12

**Authors:** Matt D G Hughes, Kalila R Cook, Sophie Cussons, Ahmad Boroumand, Arwen I I Tyler, David Head, David J Brockwell, Lorna Dougan

**Affiliations:** ^1^ School of Physics and Astronomy Faculty of Engineering and Physical Sciences University of Leeds Leeds LS2 9JT UK; ^2^ Astbury Centre for Structural Molecular Biology University of Leeds Leeds LS2 9JT UK; ^3^ School of Molecular and Cellular Biology Faculty of Biological Sciences University of Leeds Leeds LS2 9JT UK; ^4^ School of Food Science and Nutrition Faculty of Environment University of Leeds Leeds LS2 9JT UK; ^5^ School of Computer Science Faculty of Engineering and Physical Sciences University of Leeds Leeds LS2 9JT UK

**Keywords:** biomaterials, biomimetic and bioinspired materials, hierarchical biomechanics, network formation

## Abstract

The structural evolution of hierarchical structures of nanoscale biomolecules is crucial for the construction of functional networks in vivo and in vitro. Despite the ubiquity of these networks, the physical mechanisms behind their formation and self‐assembly remains poorly understood. Here, this study uses photochemically cross‐linked folded protein hydrogels as a model biopolymer network system, with a combined time‐resolved rheology and small‐angle x‐ray scattering (SAXS) approach to probe both the load‐bearing structures and network architectures respectively thereby providing a cross‐length scale understanding of the network formation. Combining SAXS, rheology, and kinetic modeling, a dual formation mechanism consisting of a primary formation phase is proposed, where monomeric folded proteins create the preliminary protein network scaffold; and a subsequent secondary formation phase, where both additional intra‐network cross‐links form and larger oligomers diffuse to join the preliminary network, leading to a denser more mechanically robust structure. Identifying this as the origin of the structural and mechanical properties of protein networks creates future opportunities to understand hierarchical biomechanics in vivo and develop functional, designed‐for‐purpose, biomaterials.

## Graphical abstract

Time‐resolved structural measurements reveal the presence of two distinct formation modes in folded protein networks: Primary Formation (purple) of the preliminary network resulting from the diffusion of protein building blocks; and Secondary Formation (dark yellow) densifying the network via the slower diffusion of high‐order cross‐linked protein oligomers formed during the primary formation, joining the network and the formation of ‘intra’‐network cross‐links.

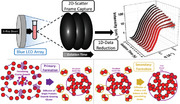

## 1 Introduction

Biomolecule networks and assemblies are exploited in both modern biomedical applications, from engineered cell scaffolds^[^ ^1^ ^]^ to drug delivery systems,^[^ ^2^ ^]^ and in processes fundamental to life, from plant cell walls^[^ ^3^ ^]^ to tendon fibers.^[^ ^4^ ^]^ This wide applicability is largely due to the hierarchical structures across length scales leading to a wide range of complex mechanical behaviors, including extraordinary strength and resilience and adaptation to environment change,^[^ ^5^, ^6^, ^7^ ^]^ e.g. the high extensibility^[^ ^8^ ^]^ and shear stiffening at low strains^[^ ^9^ ^]^ (<1%) of fibrin blood clots; and the reversible softening of actin cytoskeletons^[^ ^10^ ^]^ providing a possible mechanism for cells to respond to external stresses. Their ubiquity is twofold: i) they allow small molecules, such as proteins, to be used in the construction of large‐scale biological structures, and ii) they are crucial for the translation of single protein properties across length‐scales and time‐scales, leading to a diverse range of behavior necessary for different tissues/scaffolds. Proteins are the building blocks of these assemblies, the workhorses of the cell, performing their function through structural and mechanical changes. A beautiful example is the extracellular matrix, a 3D, dynamic network of proteins and polysaccharides that adapts to environmental conditions, such as compression and shear stress, and guide cellular organization and behavior.^[^ ^11^ ^]^ The correct self‐assembly or formation of hierarchical structures is crucial to achieve the necessary architectures and mechanics, for example fibrin blood clots, which must spontaneously form a large, fibrous, homogeneous network,^[^ ^12^ ^]^ or cell scaffolds which must form with the correct structure (e.g., the correct pore size distribution of the material)^[^ ^13^ ^]^ and mechanics (e.g., network stiffness/rigidity)^[^ ^14^, ^15^ ^]^ to promote cell growth. However, despite the ubiquity of these biopolymer networks, the mechanisms of their in situ formation remain under‐investigated. The physical mechanisms and kinetics of these assembly processes are crucial to understanding the emergent properties of these in vivo networks and creating opportunities for the design of complex viscoelastic materials.^[^ ^16^ ^]^

Time‐resolved small‐angle scattering (SAS) is a powerful technique for directly measuring the structure of a material as a function of time. Over the last several years, time‐resolved SAS has been used to monitor the formation of multiple photo‐initiated cross‐linked materials including polyether urethane commonly used in 3D print resins,^[^ ^17^ ^]^ and starch nanoparticle hydrogels^[^ ^18^ ^]^ for applications in agriculture or bioremediation.^[^ ^19^, ^20^, ^21^, ^22^, ^23^ ^]^ Additionally, it has been successful in determining the formation behavior of block co‐polymer nanoparticles in complex conditions such as under flow.^[^ ^24^ ^]^ While time‐resolved SAS is ideal for investigating the formation kinetics of materials, it has also successfully been used to understand the kinetic behavior of other processes such as ongoing chemical reactions^[^ ^25^ ^]^ or the absorption onto/into materials, including the silicification of DNA origami^[^ ^26^ ^]^ or the adsorption of water into porous silica.^[^ ^27^ ^]^ Despite the success of applying time‐resolved SAS for a range of soft matter and biological materials, it has yet to be employed to study networks constructed from folded proteins. A pioneering study by Li et al. engineered a protein hydrogel that aimed to mimic the mechanical properties of the giant muscle protein titin.^[^ ^28^ ^]^ Further studies have approximated the mechanical properties of tissues, forming highly elastic and stimuli‐responsive materials, dynamically regulating their mechanical properties and shape.^[^ ^29^, ^30^, ^31^, ^32^, ^33^, ^34^, ^35^, ^36^, ^37^, ^38^ ^]^ However, a fundamental challenge is to relate the structural and mechanical properties of an individual protein building block to the collective response of a protein network.^[^ ^39^ ^]^ Virtually nothing is known about the structure during protein network formation, severely limiting our understanding of the physics of these systems.

In 2017, we provided the first detailed structural characterization of folded globular protein hydrogels.^[^ ^40^ ^]^ Through the application of small‐angle neutron and x‐ray scattering (SANS and SAXS, respectively) we have proposed that these networks are constructed from fractal‐like clusters of cross‐linked folded proteins, which are connected by an intercluster region, populated by folded or unfolded protein.^[^ ^41^, ^42^, ^43^, ^44^ ^]^ Recent modeling approaches to understand the structure of globular protein networks have included coarse‐grained dynamic simulations which investigated the importance of cross‐link location and density on network formation^[^ ^45^, ^46^ ^]^ and on network structure as well as the effects of building block flexibility.^[^ ^47^ ^]^ Additionally, kinetic lattice‐based models have been used to investigate the importance of diffusion limited (i.e., where the formation rate limiting factor is the diffusion of the particles) and reaction limited (i.e., where the rate limiting step is the cross‐linking reaction rate) cluster aggregation in the formation of colloidal network structures.^[^ ^48^, ^49^, ^50^ ^]^ Other simulations have taken a different approach focusing on force propagation through heterogeneous cross‐linked networks of colloids^[^ ^51^ ^]^ or polymers.^[^ ^52^ ^]^ Buehler et al^[^ ^52^ ^]^ investigated the force propagation pathway through polymer networks via characterization of the convolutedness of the network (i.e., how much of the network must be explored to travel from one end to the other), finding an inverse relationship between convolutedness and connectivity. Another study^[^ ^51^ ^]^ used a similar method of “trimming” a permanently cross‐linked colloidal network to the connection path along a specific axis. This “trimmed” network was assumed to directly experience force as the network was extended along this axis and allowed for the determination of the force per colloidal particle upon external stress to the network.

Theoretical approaches have been undertaken to link the complex heterogeneous structure of networks to their mechanical properties. These approaches have included, but are not limited to: semi‐flexible polymeric theories to model the elasticity and strain stiffening of biopolymer networks,^[^ ^9^, ^39^, ^53^ ^]^ kinetic models to capture catch‐slip behavior,^[^ ^54^ ^]^ Gaussian connectivity and coordination geometry models to understand critically self‐supporting networks^[^ ^55^, ^56^, ^57^ ^]^ and colloidal modeling to capture rigidity percolation.^[^ ^58^, ^59^, ^60^, ^61^, ^62^ ^]^ Of specific relevance to this study are the works of Del Gado et al^[^ ^58^, ^59^ ^]^ and Furst et al.^[^ ^60^ ^]^ which showed that structural heterogeneity (i.e., clusters and interconnection between clusters) and structural correlations (i.e., specific attractive interaction promoting ordering) were crucial for network rigidity percolation (i.e., the formation of a self‐supporting network). These were combined with Cauchy‐Born ‐esque models^[^ ^62^ ^]^ to demonstrate that the connectivity of the clusters was crucial for the mechanical stability of the network concurring with the previously proposed rigidity network.^[^ ^58^ ^]^

In this work, we utilize time‐resolved SAXS to monitor the formation of a folded protein network constructed from bovine serum albumin (BSA) protein. In conjunction with time‐resolved SAXS experiments, we make use of shear rheology to directly probe the load‐bearing structure of the folded protein hydrogel networks. The SAXS data is combined with information gained from kinetic lattice‐based simulations and rheological characterization of the protein hydrogels to provide an integrated view of the folded protein network formation and to propose a dual process formation model.

## 2 Results

### 2.1 Selection of Model Method and System

**Figure 1:**
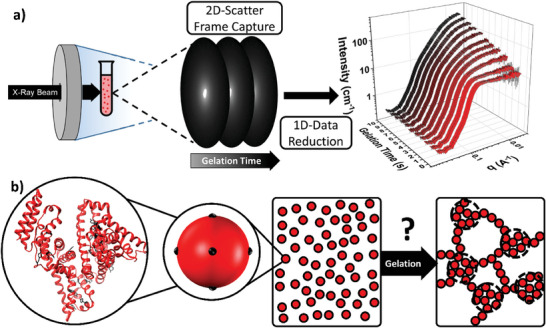
BSA hydrogels offer a model system to study the structural evolution during the formation of protein networks with time resolved SAXS. a) A schematic representation of the experiment set‐up used for time resolved SAXS of photochemically cross‐linked BSA hydrogel networks. Samples of pre‐gel BSA solutions (light red test tube) are illuminated by a bespoke blue LED rig (grey ring) to initiate gelation. Exemplar 2D and 1D scattering patterns are shown. b) BSA crystal structure with tyrosine residues highlighted in black. Schematic showing the homogeneous pre‐gel protein solution (left) where each BSA protein is represented by a red sphere with black cross‐link sites and the previously determined final network structure of BSA hydrogels (right), where fractal‐like clusters of cross‐linked BSA (red circles with dashed black ring) are connected by an inter‐cluster region of folded protein.

To determine the evolution of protein network structure, we measure structure formation during photochemical cross‐linking. Time‐resolved SAXS (**Figure** **1**a) is an ideal technique to investigate the structural evolution of biopolymer networks, due to its high flux and rapid acquisition times.

**Figure 2:**
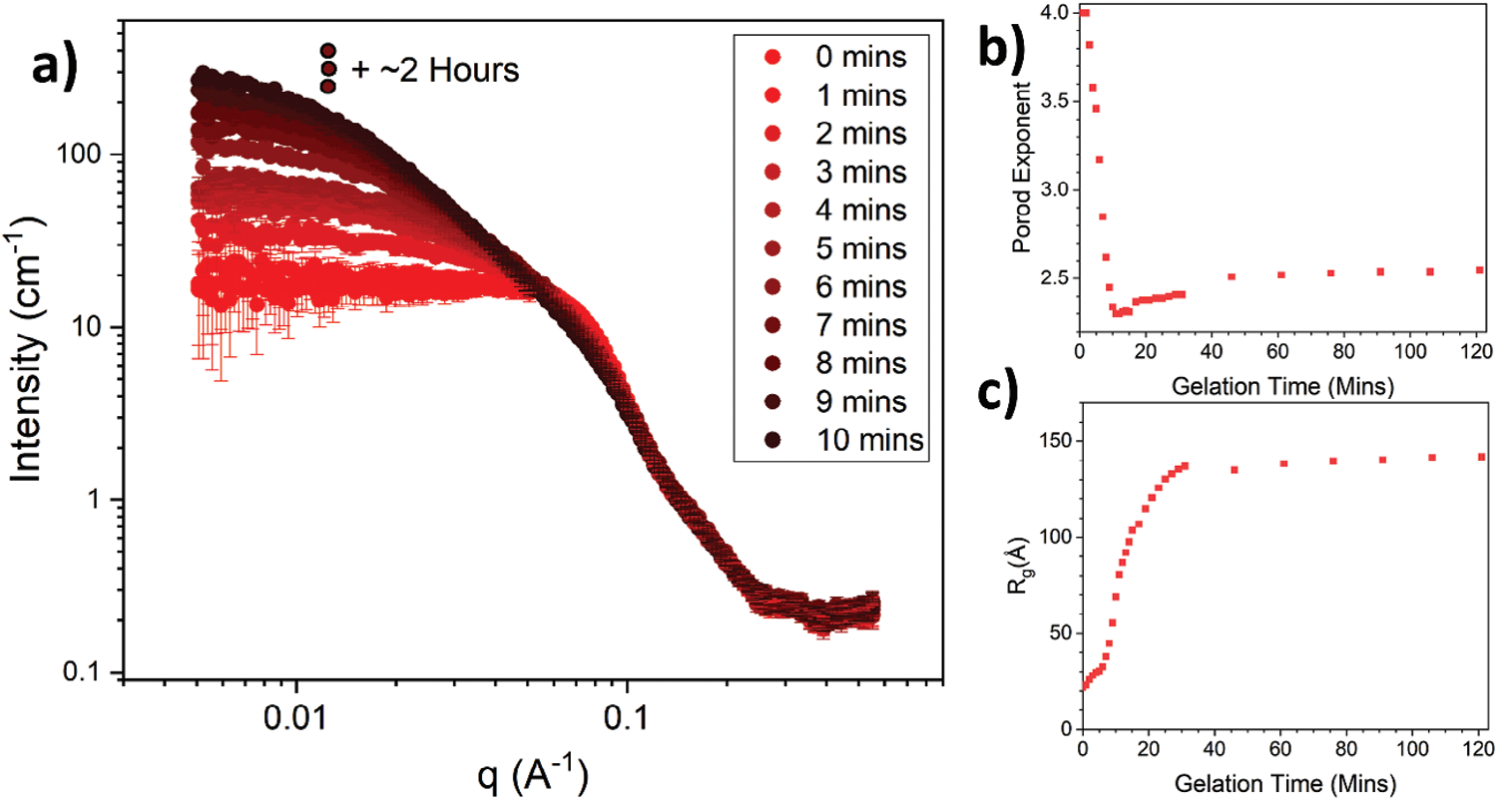
Time resolved SAXS monitors the structural formation of folded protein networks. a) SAXS curves of BSA hydrogel networks (final concentrations: 100 mg mL^−1^ BSA, 50 mm NaPS, 100 µM Ru(BiPy)_3_) as a function of gelation time. For clarity, data collected beyond 10 min and up to 2 h are not shown. b,c) The model‐independent fit parameters, Porod exponent (b) and radius of gyration, R_g_ (c), extracted from the SAXS curves in panel a) as a function of gelation time (Methods) for BSA protein hydrogel.

To study these formation mechanisms, we utilize the well‐characterized bovine serum albumin (BSA) protein (Figure 1b) hydrogels,^[^ ^2^, ^36^, ^37^, ^44^, ^63^ ^]^ as a model system. These hydrogels are formed via photo‐initiated chemical cross‐linking of tyrosine residues on the surface of the BSA protein via free‐radicalization of the tyrosine aromatic rings and subsequent tautomerization^[^ ^64^ ^]^ (Methods). Using this experimental setup, i.e., short exposure time and wide q‐range (0.005 ≤ q ≤ 0.6) with a bespoke LED light ring for the SAXS instrument (Experimental Section), we capture the evolution of the hydrogel structure over time (>2 h, **Figure** **2**a). BSA folded protein hydrogels are an ideal model system due to BSAs 18 surface accessible tyrosines, which is above the geometric limit of 4 necessary for the formation of gel networks via photochemical cross‐linking.^[^ ^65^ ^]^ Additionally, 17 disulfide bonds throughout its structure act like internal “nano‐staples”, imbuing the protein with a high resistance to force‐induced unfolding, meaning that unfolding in situ within the network is prevented and, as such, can be excluded from consideration.^[^ ^44^ ^]^

### 2.2 Structural Characterisation of Network Formation

Figure 2a shows how the scattering curves of BSA in the pre‐gel solution at 100mg mL^−1^ evolve over the first 10 min of photochemical gelation time (defined as the time the sample is illuminated by the blue light). From these curves it can be seen at gelation time, *t* = 0 the SAXS curve shows the expected shape for repulsive globular colloids at high concentrations (protein volume fraction = 7.4%), i.e., a depressed I(0) and a “peak” in the curve in the mid‐q range before a power law decrease at high‐q with an exponent of ≈−4. This scattering profile is consistent with monomeric electrostatic BSA protein at 100mg mL^−1^ previously observed with SAXS.^[^ ^66^ ^]^

The scattering curve profile changes with gelation time, most notably in the low‐q region, where we observed a dramatic increase in the intensity at low q with time. This is indicative of a growing structure forming in the system, as expected for chemically cross‐linked BSA protein. To begin to understand the evolution of the network structure over time, we perform model‐independent Guinier‐Porod fits to SAXS curves to extract the Porod exponent (Figure 2b) and the radius of gyration (Figure 2c) of the largest scattering object. The evolution of the Porod exponent (Figure 2b), gives information on how the geometry of the structure changes with gelation time. Initially, the exponent is 4, which is a characteristic of a globular object, as expected for a folded globular protein. As the gelation time increases, the Porod exponent drops rapidly, reaching a minimum at *t* ∼ 10 min. This rapid decrease in the Porod exponent is accompanied by an increase in the radius of the gyration of the largest scattering object, as shown in Figure 2c. These results suggest that larger structures are forming and that the Porod exponent is shifting from measuring the globular nature of individual proteins to a measure of the geometry of larger structures of chemically cross‐linked proteins. Porod exponents between 1 and 3 can be interpreted as a measure of the level of space‐filling (which can be thought of as how compact the structure is), with larger values being indicative of greater space‐filling (e.g., a densely packed polymer has a Porod exponent of 3, while an extended Gaussian chain has a Porod exponent of 1.67).^[^ ^67^ ^]^ Here, the minimum value of the Porod exponent is ≈2.3, suggesting that a relatively space‐filling (i.e., compact) network is formed. However, as the gelation time increases, there is a minimum in the Porod exponent data, which then slowly increases with gelation time (Figure 2b). This suggests that the network structures are becoming increasingly space‐filling over time. Note the Porod exponent and R_g_ both appear to plateau at ≈*t* = 30 min, demonstrating that the largest scattering structures are not significantly changing in geometry or size after this time and that a steady state structure has been reached. From this model‐independent scattering analysis, particularly the turning point in the evolution of the Porod exponent, it is clear there are two processes occurring in the formation of the protein network: i) an initial process that forms a less compact, sparse structure (i.e., lower Porod exponent) and; ii) a second process which increases both the overall size and the space‐filling or “compactness” of network structures.

To further explore the data, we apply a fractal structure factor model (Equation 5, Experimental Section) that describes the protein network as fractal‐like clusters of folded protein linked together by an inter‐cluster region populated by folded and unfolded proteins.^[^ ^2^, ^41^, ^42^, ^43^, ^44^, ^63^ ^]^ We extract the evolution of key structural parameters: the proportion of protein in fractal‐like clusters, p_c_; the fractal dimension, D_f_, which is higher for more compact fractal‐like clusters containing a greater “density” of cross‐linked protein; and the characteristic length, ξ, which is related to the size of the clusters.

**Figure 3:**
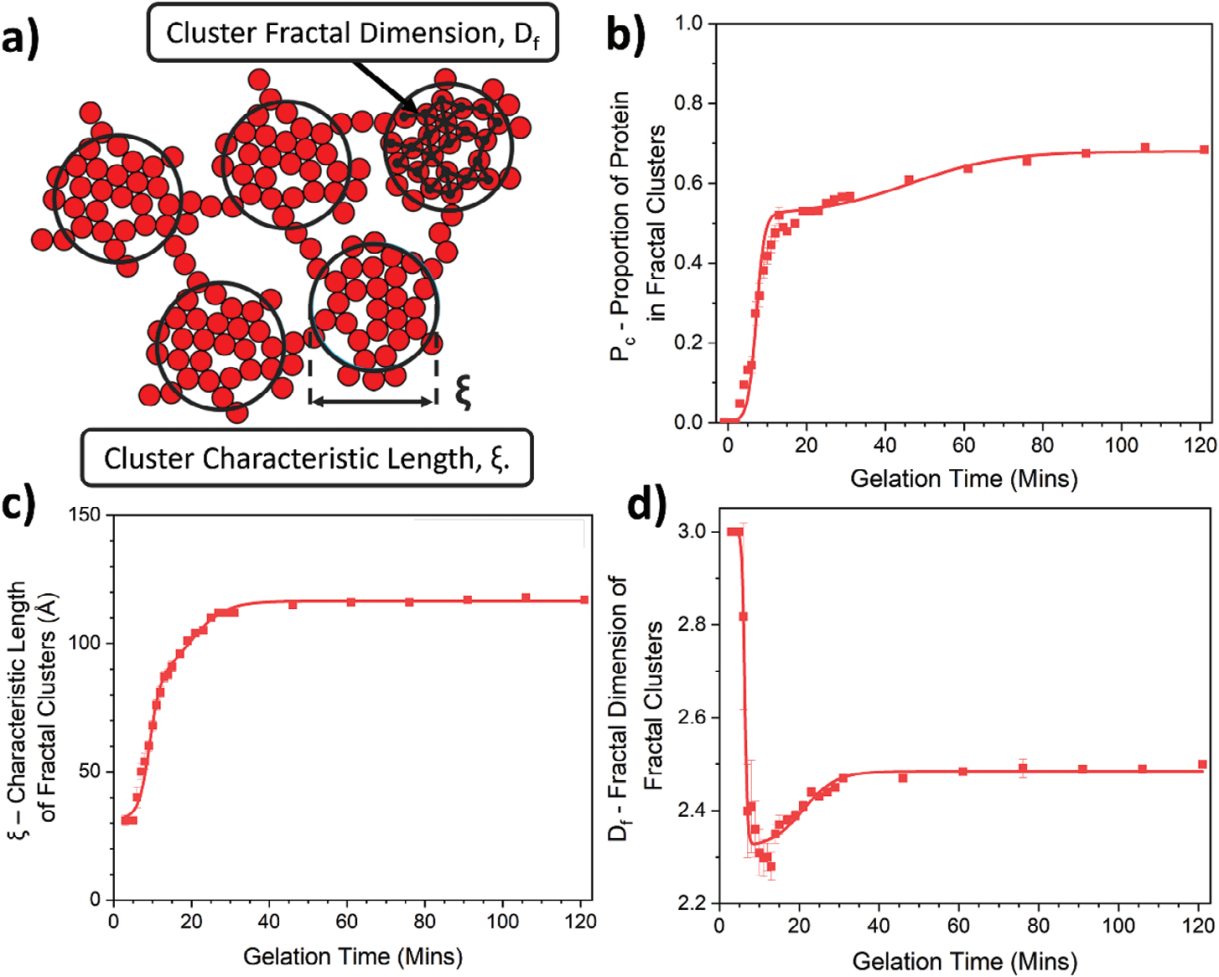
Time resolved SAXS reveals the evolution of key structural parameters during folded protein network formation. a) Schematic showing the previously determined final network structure of BSA hydrogels (red circles), where fractal‐like clusters of cross‐linked BSA (identified by black rings) are connected by an inter‐cluster region of folded protein. b,c,d) The fractal cluster model dependant fit (Equation 5) parameters; proportion of folded protein in fractal clusters, *P_c_*,(b); the characteristic lengthscales of the fractal‐like clusters, ξ (c); and the fractal dimension of the fractal‐like clusters, D_f_,(d), extracted from the SAXS curves in Figure 2a as a function of gelation time for BSA networks. The solid lines show fits using Equation (1).

**Figure 4:**
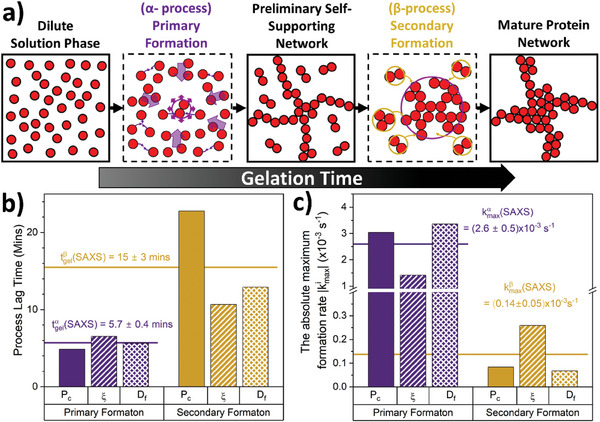
Folded protein networks are formed by a dual formation process consisting of a fast initial primary formation and a subsequent slower formation. a) Schematic showing the transition from free protein in solution through putative self‐supporting network phase to mature protein network, highlighting the two formation processes. The first α‐process called the Primary Formation (purple) which is the formation of the preliminary network resulting from the diffusion of protein building blocks. The second β‐process called the Secondary Formation (dark yellow) due to the slower diffusion of high‐order cross‐linked protein oligomers formed during the primary formation, (depicted here as dimers and trimers for simplicity) joining the network as well as the formation of “intra”‐network cross‐links both resulting in densification of the network. b) Extracted lag times from Figures 3b–d, labeled P_c_, ξ, D_f_ respectively, for both the primary (purple) and secondary (dark yellow) formation. The solid lines represent the average lag time for each formation mechanism as extracted from SAXS data. c) Extracted normalized absolute k_max_ values from Figure 3b–d, labeled P_c_, ξ, D_f_ respectively, for both the primary and secondary formation. The solid lines represent the average normalized k_max_ for each formation mechanism as extracted from SAXS data.

The extracted parameters, p_c_, ξ, and D_f_ are shown in **Figures** **3**b,c,d, respectively. As expected, we observe an increase in the proportion of protein in clusters, p_c_, over time as more proteins join clusters within the network over time. After an initial lag period of ≈5 min, there is a sharp increase in p_c_. This sharp growth in the proportion of proteins in large, cross‐linked protein clusters, lasts until t ∼ 8 min. After the rapid increase in p_c_ there is a slower growth rate which plateaus at a p_c_ value of 0.670 ± 0.004. In conjunction with the time evolution of p_c_, we observe a similar two‐phase increase in the correlation length (Figure 3c) of fractal‐like protein clusters, with an initial rapid increase in cluster size followed by a slower growth to a final plateau value of 116.5 ± 0.3 Å. The evolution of the cluster size with gelation times shows that clusters increase in size with time. The similarity between evolution profiles of p_c_ and ξ makes sense as the more proteins that cross‐link into the cluster the larger the cluster will grow in size to accommodate the additional protein. Interestingly, while p_c_ and ξ increase monotonically with gelation time, we observed a non‐monotonic evolution of D_f_ with gelation time (Figure 3d), i.e., the fractal dimension initially rapidly decreases from a value of three down to a minimum of value of 2.28 ± 0.03 at a time, *t* = 13 min. At this minimum point, the trend reverses and D_f_ slowly increases up to a plateau value of 2.48 ± 0.01, following a profile comparable to the Porod exponents in Figure 2b, as expected. Taken together, the scattering data suggest an initial configuration of the network into smaller (characteristic size of ≈90 Å, shown by the ankle in Figure 3c at t ≈13 min) dendritic (D_f_ (Minimum) = 2.28 ± 0.03) percolating clusters (**Figure** **4**a), i.e., forming a system spanning network (previous rheology data on BSA hydrogels^[^ ^44^ ^]^ demonstrate the network is mechanical self‐supporting after ≈5 min). These percolated clusters continue to grow more slowly (up to an end‐point value of 116.5 ± 0.3 Å) after the initial rapid growth and importantly begin to increase in density (end point D_f_ value = 2.48 ± 0.01). Interestingly, this increase in cluster density continues up to gelation times of ≈105 min (Figure 3c), whereas the increase in cluster size plateaus at gelation times of ≈30 min. This shows that at longer gelation times, i.e., t >30 min, there is an increase in the density of fractal‐like clusters which is not accompanied by an increase in their size. This suggests that there is a process occurring at gelation times >30 min which is increasing the density of clusters without altering the overall size of the clusters. Densification of the clusters begins at gelation times of ≈15 min and occurs more rapidly (Figure 3d) while clusters are growing in size (Figure 3c) and continue more slowly after clusters no longer increase their size. This suggests that the densification of clusters is a complex process comprised of two mechanisms: i) a process where new building blocks cross‐link into clusters increasing their density and size; and ii) a process where clusters form additional cross‐links within themselves causing an increase in density with a negligible change in size. To fully disentangle the complex formation of these protein hydrogel networks, the results in Figure 3 must be further interrogated.

### 2.3 Dissection of Network Formation Mechanisms

To investigate the kinetics of the dual phase formation mechanism, we compare the lag time and maximum formation rate (the fastest rate at which the process occurs) of both the α‐primary formation and β‐secondary formation. To do this we fit the extracted structural parameters (Figure 3b–d), with a constructed empirical equation, in which each formation mechanism is modeled as a sigmoid (Equation 1):

(1) $$x\left(t\right)=\left(x\left(\infty \right)-x\left(0\right)\right)\cdot \left(\frac{\alpha }{1+e^{-k^{\alpha }\left(t-t_{0}^{\alpha }\right)}}+\frac{\beta }{1+e^{-k^{\beta }\left(t-t_{0}^{\beta }\right)}}\right)+x\left(0\right)$$

where x is the one of the extracted parameters p_c_, ξ, or D_f_; x(0) and x(∞) are the initial and end‐point values of the extracted parameter; α and β are the proportion of the primary and secondary formation processes, respectively, such that α + β = 1; k^α^ and k^β^ are related to the rate of sigmoidal growth for the two formation processes (primary and secondary); and t_0_^α^ and t_0_^β^ are the midpoints of the sigmoids modeling the alpha and beta formation processes. Fitting Equation 1 we can use the determined parameters to calculate the lag time for the i^th^ process (Equation 2), t_gel_^i^:

(2) $$t_{gel}^{i}=t_{0}^{i}\;-\frac{2}{k^{i}}$$

Similarly, the max formation rate for the i^th^ process, K_max_^i^, is

(3) $$K_{max}^{i}=\frac{i\cdot k^{i}}{4}$$

Derivations of Equations (2) and (3) can be found in the supplementary information. Figure 4b,c show the extracted lag times and maximum formation rates for the alpha and beta formation processes, determined from each of the extracted scattering parameters, P_c_, D_f_, and ξ.

We take the average of the SAXS lag times (Figure 4b) and maximum formation rates (Figure 4c) determined from each structural parameter (p_c_, D_f_, and ξ) extracted from SAXS data. Figure 4b compares the average SAXS lag times of the primary formation, $t_{gel}^{\alpha }$(SAXS), and secondary formation, $t_{gel}^{\beta }$(SAXS), showing that $t_{gel}^{\beta }$ (SAXS) > $t_{gel}^{\alpha }$ (SAXS) (i.e., primary formation occurs before secondary formation) suggesting that secondary formation is dependent on the α‐primary formation. Coupled with the difference in SAXS lag times, Figure 4c shows that there is a 19‐fold reduction in the max formation rate of the secondary formation compared to the primary formation (i.e., $k_{max}^{\alpha }$(SAXS) ≈19∙$k_{max}^{\beta }$ SAXS)). Based on the results of both the model independent and model dependent analyses we propose a dual formation mechanism (Figure 4a): (i) Primary formation characterized by rapid cross‐linking of monomeric protein to form percolating clusters leading to a dendritic spanning network; and (ii) secondary formation characterized by an increase in the size and density of the percolated cross‐linked clusters of folded protein within the network. The slower growth of secondary formation as well as the increase in size and density of the clusters may be due to the slower diffusion of cross‐linked aggregates formed during the primary phase (e.g., dimers, trimers etc.) that join the network after gelation, but could also be due to additional intra‐cluster cross‐links formed when thermal excitation of network elastic modes bring different protein branches into contact, leading to densification as observed in fibrin networks^[^ ^68^, ^69^, ^70^ ^]^ and simulations of colloidal networks.^[^ ^45^ ^]^ These potential mechanisms will be explored in the simulations below.

### 2.4 Computational Modelling of Protein Network Formation

To test the feasibility of the conjecture that the dual structural formation mechanisms seen in the experimental SAXS data can be purely driven by the diffusion of proteins and subsequently protein oligomers, we performed coarse‐grained simulations of network formation in which proteins are represented as colloid‐like units that translationally diffuse on a 3D periodic lattice. Cross‐linking is modeled by permanently joining immediately‐adjacent units with a reaction probability, R, per unit simulation time step (defined as the time for a monomeric simulated protein to diffuse its own diameter). The simulated diffusion probability (i.e., the diffusion constant) is inversely related to cluster size, i.e., monomers diffuse faster than dimers which diffuse faster than trimers, and so on. Importantly, networks formed within this kinetic model have no elasticity, thus any agreement with scattering data can only be attributed to the assimilation of cross‐linked aggregates post‐gelation, and not any mechanism involving network deformation.

**Figure 5:**
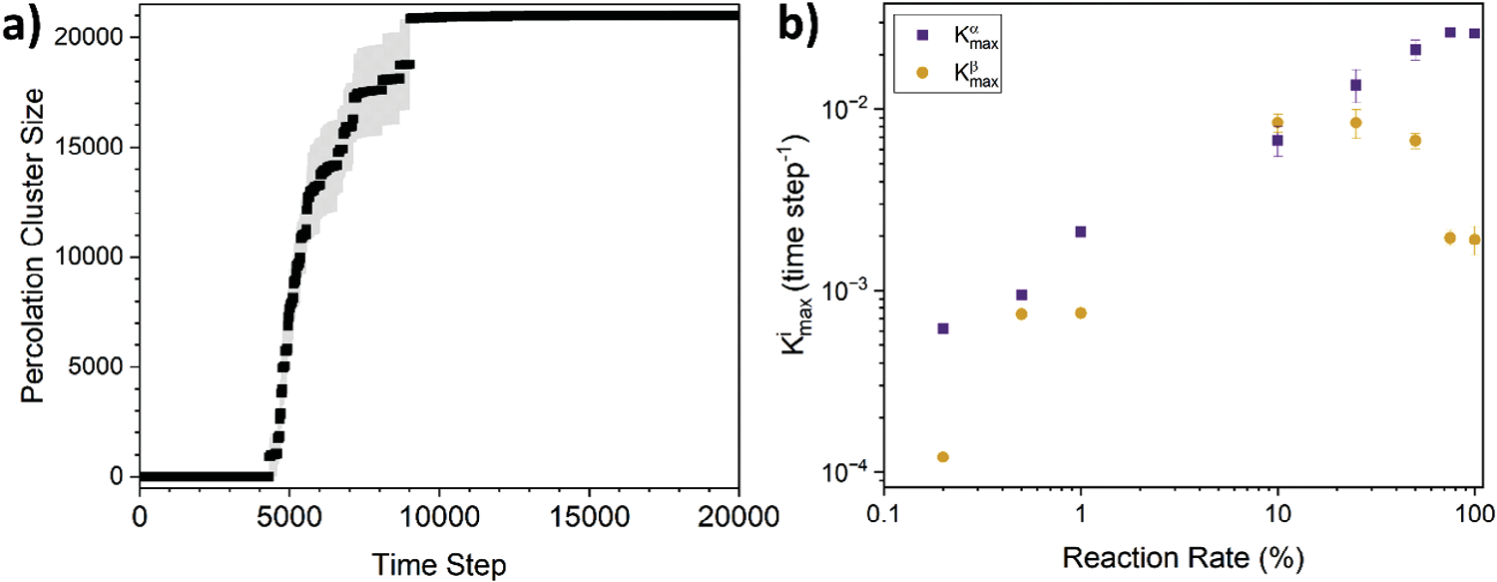
Coarse‐grained simulations reveal that primary and secondary formation are driven by diffusion of monomers and oligomers, respectively. a) An example raw simulation data curve of the number of “protein” monomers in the percolating cluster over time, averaged over 10 simulation runs, of an 8% volume fraction system with a monomer‐monomer reaction probability *R* = 0.2%. b) Extracted max fundamental formation rate for the α‐formation (purple squares) and β‐formation (dark yellow discs) phases extracted using Equations (1) and (3) as a function of simulated reaction rate R.

**Figure** **5**a shows an exemplar evolution in the size of the simulated percolated network at a reaction probability of 0.2%. The time of network percolation is identified as when, a cross‐linked cluster (typically the largest) in the system first spans the simulation box in all three dimensions, which is comparable to the lag time measured in experiments. After this time, the growth of the percolated network is quantified by the number of monomer units contained within the spanning cluster. The curve in Figure 5a shows a similar profile to that observed for the time evolution of p_c_ (Figure 3b), i.e., a lag phase followed by an initial rapid increase in the number of proteins in the percolated network and finally a slower increase before reaching a plateau. This slower late phase can be interpreted as evidence for a two‐phase formation process, suggesting that the underlying driver of the primary and secondary formation observed in the SAXS data results from the diffusion of monomers and the subsequent diffusion of larger aggregates, respectively. Fitting the simulated growth curves (Figure 5a; Figure S1a, Supporting Information) with Equation (1), we can extract the maximum fundamental rate of both processes (Equation 3) over a range of reaction rates *R* = 0.2% to *R* = 100%. Figure 5b shows how the α‐primary and β‐secondary formation rates vary as a function of reaction rate. The validity of the fitting process (Figure S1b, Supporting Information) confirms that the assimilation of freely diffusing cross‐linked oligomers post‐gelation is a viable mechanism for the two‐phase formation scenario inferred from the scattering data. Additionally, while $k_{max}^{\alpha }$ (sim) increases monotonically with reaction rate, we observe a maximum in $k_{max}^{\beta }$ (sim) at a reaction rate of approx. *R* = 20%. This reaction rate coincides with the crossover between diffusion limited cluster aggregation (DLCA) to reaction limited cluster aggregation (RLCA) observed previously.^[^ ^49^ ^]^ Previous rheological characterization of BSA hydrogel networks has suggested that at low lamp intensities (<10 mW cm^−2^) the system is in the reaction limited cluster aggregation regime^[^ ^63^ ^]^ (2.8 mW cm^−2^ is the lamp intensity used in this study). From Figure 5b, as the reaction rate decreases below 10% the separation between the $k_{max}^{\alpha }$(sim) and $k_{max}^{\beta }$ (sim) increases, where at 0.2% reaction rate the ratio $k_{max}^{\alpha }$ (sim)/ $k_{max}^{\beta }$ (sim) is ≈5. While this does not exactly match the ratio of the rates observed in SAXS ($k_{max}^{\alpha }$ (SAXS)/ $k_{max}^{\beta }$ (SAXS) ≈19), it is consistent with the experiments being deep into the reaction‐controlled regime.

Previous analysis of these simulations found an increase in fractal dimension (ΔD_f_(sim) = 0.13) of the simulated network (at a simulated reaction rate of 0.2%) from the percolation point to the final network configuration, i.e., during the secondary formation. However, the experimental increase in D_f_ between the minimum and final value ((ΔD_f_(exp) = 0.200 ± 0.03) is larger than those observed in these simulations. Therefore, while this modeling suggests that the main underlying mechanism of β‐secondary formation is the kinetic assimilation of diffusing oligomers into the network post‐gelation, the discrepancy in the change of fractal dimension suggest mechanisms involving network deformation, absent in this kinetic model (but present in dynamic simulations^[^ ^45^ ^]^), are also contributing to secondary formation.

### 2.5 The Presence of Dual Formation Modes in Network Mechanics

**Figure 6:**
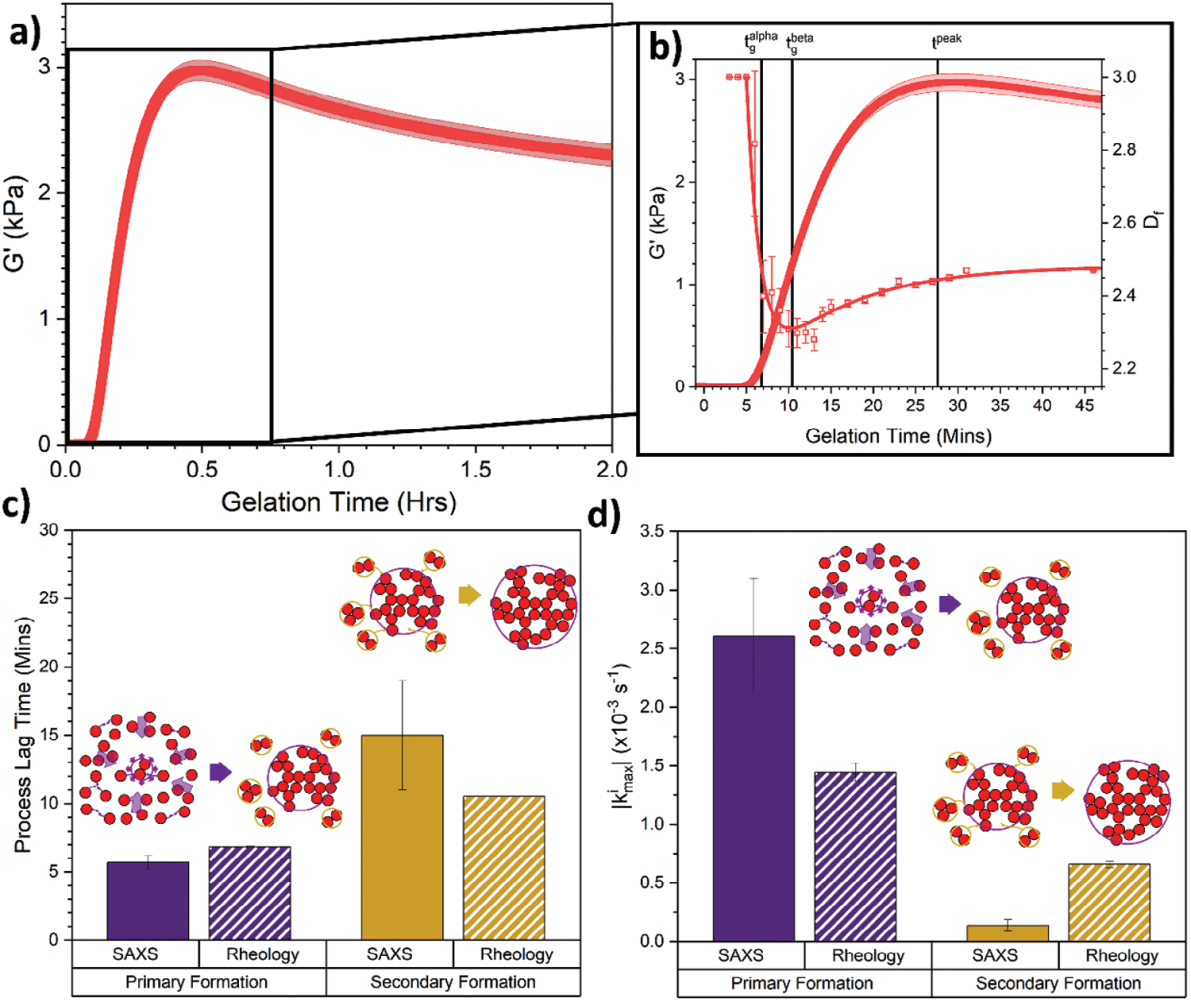
Rheology reveals a dual mechanism formation process in the mechanical formation of a self‐supporting folded protein network. a) Gelation curves, showing storage modulus as a function of gelation time of BSA hydrogels. Illuminated at *t* = 0 h till *t* = 2 h. b) Magnification of BSA gelation curves (solid symbols) in panel a between *t* = ‐1min to *t* = 30 min, with the evolution of the fractal dimension (open symbols) extracted from SAXS overlayed (Figure 3c). Vertical solid black lines represent the key lag times, $t_{gel}^{\alpha }$ and $t_{gel}^{\beta }$ extracted from the rheological data (Methods). The solid red lines represent the fit to the evolution of the fractal dimension extracted from SAXS data (Equation (5), Methods). c) Extracted lag times from both SAXS (Figure 4b) and rheology data for both the primary (purple) and secondary (dark yellow) formation. Inserts show schematics for the proposed mechanisms. d) Extracted normalized absolute k_max_ values from both SAXS (Figure 4c) and rheology for both the primary and secondary formation. Inserts show schematics for the proposed primary and secondary formation mechanisms, where the preliminary network forms from the diffusion of monomer protein building blocks (Primary formation), followed by the slower diffusion of high‐order cross‐linked protein oligomers (depicted here as dimers and trimers for simplicity) cross‐linking into the preliminary network (Secondary formation).

Time‐resolved rheology experiments were employed to investigate the presence of the primary and secondary formation in the mechanics of the folded protein hydrogel network. Using a previously developed LED rig^[^ ^63^ ^]^ for the in situ photochemical gelation of folded protein hydrogels on the rheometer, we monitored the mechanical formation of BSA hydrogels. **Figure** **6**a shows how the storage modulus, G’, (a measure of the hydrogel network elasticity) at a fixed frequency evolves as a function of gelation time for the BSA hydrogel networks.

These curves show an initial lag phase followed by a sharp increase in G’ rising to a peak, before relaxing down to a plateau value. This relaxation behavior during the gelation process has previously been observed in the gelation of BSA hydrogels. Previous studies^[^ ^43^, ^44^ ^]^ have directly studied this relaxation to a final G’(*t* = ∞) value, using circular dichroism spectroscopy (CD). CD monitored the folded structure of mechanical robust (i.e., with disulfide staples preventing unfolding) and force labile (i.e., without disulfide bonds) BSA over time post‐gelation and found that there is significant (∼ 30%) unfolding of the force labile BSA during the relaxation phase.^[^ ^44^ ^]^ This demonstrated that the relaxation behavior in folded protein networks is due, in part, to the unfolding of protein domains in the network. Interestingly, relaxation behavior is still observed in native BSA hydrogels (which due to disulfide bonds are unable to unfold) suggesting that unfolding is only one component of the relaxation behavior, the other was attributed to be due to reconfigurations of building blocks within the network. In this work we focus on the initial increase in G’ to directly probe the formation of load‐bearing structures in the system. Closer inspection of the sharp increase in the gelation curve suggests there are two regimes (and hence two formation modes) in the rising phase, similar to the SAXS data and kinetic simulations: a faster formation centered at *t* ∼ 8 min and a slower growth at *t* = 16 min. To extract values for both the formation modes simultaneously we modify a previously used fitting function^1^ (Equation 7) which models the evolution of G’ as the product of two functions: i) a single mode formation factor (Equation 8) which describes the sharp growth in G’ during photochemical cross‐linking; and ii) the relaxation factor (Equation 9) which models the decrease in G’ to a plateau value at later times. Here we replace the previously used single mode formation factor with the same two mode, double sigmoidal expression used for the scattering data (Equation 1). From the fitting of this modified Equation (7) we can then extract the lag times of both formation modes (Equation 2). These lag times for the primary α formation, t^α^_gel_(Rheo), and the secondary β formation, t^β^_gel_(Rheo), are annotated on the rheology gelation curve overlaid with the fractal dimension D_f_ (as determined from scattering) in Figure 6b. Additionally, the values with errors are shown in Figure 6c alongside the lag time values extracted from SAXS data. Note that the values of the α‐primary formation are consistent with the lag time value extracted using the conventional intersecting lines methods (Figure S2, Supporting Information), demonstrating our bespoke fitting method obtains accurate results (Equations 1 and 7). From Figure 6b we can see that extracted rheological lag times, $t_{gel}^{\alpha }$(Rheo) and $t_{gel}^{\beta }$(Rheo), coincide with the key features of the evolution of D_f_ with time, specifically: $t_{gel}^{\alpha }$ (Rheo) coincides with the initial rapid decrease in D_f_, while $t_{gel}^{\beta }$(Rheo) coincides well with the minimum in D_f_ prior to the slow increase in D_f_ at later times. This suggests that there is a link between the structural formation and the mechanical response of the network over time, as one would expect. The presence of two formation modes in the rheology data and the strong agreement between the lag times extracted from SAXS and rheology (Figure 6c) suggests that the dual formation model we proposed is not only crucial for the formation of the hydrogel architecture but also for the formation of the rigidity network of the protein network. Figure 6d compares the max fundamental formation rates extracted from both SAXS and rheology. In both cases the α‐primary formation relative rate is greater than that for the β‐secondary formation, i.e., $k_{max}^{\beta }$(Rheo) <$k_{max}^{\alpha }$ (Rheo) and similarly for the scattering rates. However, the absolute values significantly differ. The rheology α‐primary formation rate is approximately half the scattering value, suggesting that a significant fraction of proteins connected to the incipient percolating cluster do not contribute to full system mechanical response. This concurs with known results from rigidity percolation, where it has been established that forces propagate through a stress‐bearing backbone that exists within (but is smaller than) the percolating cluster.^[^ ^71^, ^72^ ^]^ For example, forces will not propagate through regions of the network only connected to the backbone at a single attachment point, so such “dangling” regions will not contribute to the rheology but will still contribute to the scattering signature. Conversely, the rheology β‐secondary rate is much greater than that for scattering, suggesting that small structural changes arising after gelation can substantially stiffen the network. Putative mechanisms include thermally‐activated elastic deformations bringing different network regions into proximity (e.g., rotation of the aforementioned ‘dangling’ regions), permitting new cross‐links to form on the stress‐bearing backbone and additional constraints to the network mechanical response. Similarly, freely‐diffusing protein aggregates may attach to the network and create a new bridge between nearly‐touching network branches, similarly introducing constraints and lowering network flexibility. Our kinetic modeling above suggests both classes of mechanism are required to fully describe secondary formation processes, but additional, dynamic modeling is required to quantitatively capture both the structural formation, as well as force propagation and bulk mechanics.

## 3 Discussion

The formation of biomaterials is a crucial process to designing and constructing materials fit for purpose. Here, by combining time‐resolved SAXS and computational modeling with complimentary rheological experiments we disentangle the complex formation behavior of a model protein network. We identify two distinct formation phases that build the protein network architecture and rigidity: i) an initial primary formation which is characterized by the diffusion and cross‐linking of protein monomer into a dendritic, system spanning, self‐supporting network consisting of percolated cross‐linked clusters; and ii) a subsequent secondary formation characterized by additional cross‐linking due to internal network deformation modes bringing regions into contact for additional cross‐linking, or the incorporation of freely‐diffusing aggregates into the system‐spanning cluster, resulting in growth and densification of the clusters already embedded in the network. It should be noted that the present dual formation mechanism was observed at a single concentration of BSA (100 mg ml^−1^) and ionic strength (≈0.2m). At this concentration it is expected that BSA in solution has a screened Coulombic structure, which would limit self‐association into oligomers pre‐gelation.^[^ ^66^ ^]^ At higher ionic strengths BSA has been observed to oligomerise in solution.^[^ ^66^ ^]^ This suggests that at high ionic strengths an increased prevalence of oligomers may be present and a more dominant role for the secondary formation mechanism. This may provide an interesting route to explore the structural evolution of protein networks which are manipulated through control of electrolytes. A similar formation has been observed in a dilute solution of large colloidal particles (≈3µm), in which fractal cluster initially form and undergo a densification at later times.^[^ ^73^ ^]^ This suggests that the dual modal model of folded protein network formation proposed in this work is applicable both to other colloidal systems and across a range of building block size, i.e., from nms to µms.

By performing both time‐resolved SAXS and shear rheology we were able to separately and directly probe the evolution of the network architecture and the load‐bearing network of the folded protein hydrogels, respectively. The formation rates extracted from SAXS and rheology (Figure 6c,d), were found to agree, suggesting there is a close link between the network architecture and rigidity. Additionally, a maximum was observed in the secondary formation rate extracted from simulations of the kinetic lattice‐based model, likely due to a switch from DLCA to RLCA.

Previous experimental and modeling studies^[^ ^49^, ^63^ ^]^ have shown that cross‐linking reaction rate modulates the mechanics and structure of folded protein networks, with networks formed at high intensities exhibiting more dendritic structures with higher mechanical rigidity.^[^ ^63^ ^]^ Additionally previous computational modeling reveals that reaction rate alters their simulated formation kinetics (e.g., higher reaction rates, shorter percolation times).^[^ ^49^ ^]^ Both experimental and modeling studies showed reaction rate‐dependent changes that are consistent with a transition between diffusion‐ and reaction‐limited assembly, and corresponding changes to the viscoelastic response of the protein networks. The results presented here combined with previous literature suggest reaction rate offers a facile method for varying gel properties at a fixed volume fraction and a route to further understand the in situ formation mechanisms of protein networks.

Previous work by Furst et al.^[^ ^60^ ^]^ and del Gado et al.^[^ ^58^ ^]^ have used Cauchy‐Born models to explain the mechanics of their colloidal systems at the level of clusters, deriving

(4) $$G^{\prime }=2Zn_{c}k\langle r^{2}\rangle$$

in which Z is the average coordination of a cluster, n_c_ is the cluster number density, k is the spring constant for the linkages between clusters, and r is the characteristic cluster size. From our SAXS data and our previously published method^[^ ^44^ ^]^ we can determine r and n_c,_ and the variation of G’ is immediately available from our rheology data. Therefore, using these calculated values and Equation (4) we can determine how the product of cluster connectivity and inter‐cluster stiffness is predicted to vary over time.

**Figure 7:**
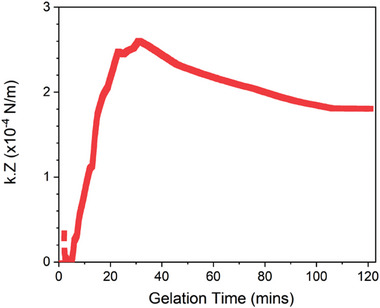
Cauchy‐Born models currently cannot fully model folded protein networks. The product of the cluster coordination, Z, and the spring constant of the cluster‐cluster connection, κ, as a function of the gelation time. Here, κ.Z is calculated as $\frac{G^{\prime }}{2n_{c}\left\langle r^{2}\right\rangle }$.

**Figure** **7** shows the results of this cluster‐level Cauchy‐Born model. The values for the product *k*∙Z are small over all gelation times, with an end point value of (1.8 ± 0.03)x10^−4^ N m^−1^. If we assume the Z is a positive number between 4 (minimum coordination for self‐supporting networks) and 12 (maximum coordination of spheres), then k is on the order of 10^−5^ N m^−1^. These values are significantly below the estimated values for protein stiffness from quasi‐elastic neutron scattering, order of 10^−1^ N m^−1^.^[^ ^74^ ^]^ This suggests that current models of clustered colloidal networks are not able to fully capture the translation of protein stiffness across length scales in hierarchical protein networks. This is supported by the difference we observe in the formation rates extracted via SAXS and rheology, which suggest that the mechanical contribution of the protein monomers (during primary formation) and oligomers (during secondary formation) is dependent upon when, where, and how they join and link into the network. Despite the discrepancy between our estimate *k* value and the estimate stiffness of folded proteins, the profile of the product *k*Z matches extremely well with the profile of our rheological gelation curves. In particular, we observe a sharp increase in *k*Z after a lag phase be peaking and decreasing to a plateau value (Figure 7). The observed decrease in *k*Z after t ≈ 30 min aligns well with the relaxation behavior observed in the rheological gelation curves, suggesting the origin of the relaxation is found in the product *k*Z. Since our hydrogel system is constructed via covalent photochemical cross‐linking, the coordination number Z is unlikely to change, meaning the relaxation behavior is likely due to changes to the interconnecting stiffness value *k*. Since in our system *k* is related to the stiffness of folded protein linking clusters together, we speculate this means that the relaxation behavior observed rheologically (Figure 6a) is not due to network‐level reconfiguration of the network but rather due to changes on the protein level, e.g., protein domain weakening. Our work here highlights the need for more development of theoretical and computational models able to determine the location and importance of building blocks that are key to the rigidity network and how force is propagated through them. New computational and theoretical models such as Buehler et al recent work^[^ ^52^ ^]^ will help us to understand the cross‐length scale translation of mechanical properties in protein networks.

The dual formation model proposed in this work has implications on understanding how in vivo protein networks form, for example fibrin blood clots. This work suggests that fibrin blood clots form first by the primary formation percolation of an initial network of proto‐fibers before secondary formation takes place leading to bundling and the production of mature fibers. Such a model of fibrin formation has been previously suggested from simulations of patchy rod‐like particle aggregation by Rocco et al.^[^ ^70^ ^]^ This is also consistent with our previous study^10^ which demonstrated that the assembly of fibrin proto‐fibers into a self‐supporting network is the initial step in clot formation as opposed to bundling.

The BSA protein building block used in this study contains 17 structural disulfide bonds which infer mechanical robustness and limit protein unfolding. The mechanically robust BSA protein was photochemically cross‐linked to create a percolating colloidal‐like network of folded proteins. In the present study we find evidence for a dual formation mechanism for protein network formation. It is interesting to consider the possible universality of this model for other protein systems. Our previous studies of force labile proteins have demonstrated that protein unfolding is a critical determinant of network structure and mechanics.^[^ ^44^ ^]^ Therefore, we expect that future in situ studies of protein network formation will reveal rich differences in the formation mechanisms which are governed by the force lability of the protein building block and protein unfolding during network formation. Furthermore, previous studies have shown a complex interplay between the thermodynamic stability of the protein and how it directly governs protein network properties while the protein unfolding kinetics limits the tunability accessible via the thermodynamic stability.^[^ ^42^ ^]^ The methods and analysis presented in this study are therefore an important first step toward uncovering the dynamic assembly of more complex protein networks, including those which are responsive to chemical and mechanical perturbations.

## 4 Experimental Section

### Materials

Bovine serum albumin (heat shock fraction, protease free, fatty acid‐free, and essentially immunoglobulin free), tris(2,2′‐bipyridyl)dichlororuthenium(II) hexahydrate (Ru(BiPy)_3_), sodium persulfate (NaPS), sodium phosphate dibasic, and sodium phosphate monobasic were obtained from Sigma‐Aldrich, and used without further treatment.

### Sample Preparation

As previously published,^[^ ^2^, ^44^, ^63^ ^]^ hydrogel samples are prepared by mixing in a 1:1 ratio a 200 mg mL^−1^ stock of either BSA protein and 2 × cross‐link reagent stock for final protein and reagent concentrations of 100 mg mL^−1^ BSA, 50 mm NaPS, and 100 µm Ru(BiPy)_3_.

### Small Angle X‐Ray Scattering (SAXS)

SAXS measurements were conducted on a Xeuss 3.0 offline SAXS instrument (Xenocs Inc., France) using a gallium rich alloy liquid metal jet x‐ray source, (Ga K_α_ = 9.2keV (1.3 Å)) (Excillum, Sweden). Samples were loaded into 1.48mm path length capillary tubes (Capillary Tube Supplies Ltd.), sealed with manuscript sealing wax. Sealed capillary tubes were loaded into a Xenocs Peltier capillary holder and held at a constant temperature of 20 °C. The detector was run at two distances from the sample at 4.5m and 0.5m giving the investigated q‐range of 0.005 to 0.5 Å^−1^. Samples were photo‐initiated and gelated in situ in the x‐ray sample chamber using a bespoke blue LED lighting rig (Figure S2, Supporting Information, Acknowledgements). 2‐D SAXS patterns were recorded on an Eiger2 R 1M detector (Dectris, Switzerland),. Silver behenate (a = 58.38 Å) was used to calibrate the SAXS data and glassy carbon calibration was performed to convert data to absolute intensities. SAXS curves were acquired over multiple frames. Frame times began at 1 min (52s acquisition time + 8s processing time) for the first 15 min of gelation, then increased to 2 min (108s acquisition time + 12s processing time) until the gelation time *t* = 31 min. After 31 min of the sample being illuminatied by the blue light the frame time was set at 15 min (850s acquisition time + 50s processing time). Note, control experiments were done to ensure that there was no radiation damage to the protein, nor did x‐rays alone activate the photochemical cross‐linking (Figure S4, Supporting Information). SAXS data were processed using the DAWN software.^[^ ^75^ ^]^

### SAXS Analysis

SAXS curves obtained were fitted using SASview (http://www.sasview.org). Model‐independent fits were performed and consisted of a Guinier‐Porod fit^[^ ^11^ ^]^ over the entire data range, to extracted the Porod exponent and the radius of gyration at specific time point for the evolving system. Model dependant fits were performed in accordance with Equation (5).

(5) $$I\;\left(q\right)=Scale\cdot P\left(q\right)\left[\left(1-p_{c}\right)+p_{c}S\left(q\right)\right]+Background$$

here, Scale is a scaling factor, p_c_ is the proportion of folded protein in clusters, P(q) is a Guinier‐Porod form factor^[^ ^76^, ^77^ ^]^ to model the general size and shape of the folded protein, and S(q) is a fractal structure factor,^[^ ^78^ ^]^ defined as:

(6) $$S\;\left(q\right)=\;\frac{D_{f}\Gamma \left(D_{f}-1\right)}{{\left[1+\frac{1}{{\left(q\xi \right)}^{2}}\right]}^{\frac{D_{f}-1}{2}}}\cdot \frac{sin\left[\left(D_{f}-1\right)\left(q\xi \right)\right]\;}{{\left(qR_{0}\right)}^{D_{f}}}$$

where D_f_, ξ, and R_0_ are defined as the mass fractal dimension, correlation length, and minimum cutoff length scale defined by the form factor, respectively.

### Rheometry

Mechanical characterization experiments of BSA hydrogel samples were performed on an Anton Paar MCR 302 stress‐controlled rheometer (Anton Paar GmbH, Austria) in parallel plate configuration (with a plate diameter of 8 mm). Samples of pre‐gel solutions were added to the parallel plate with a gap height of 1.48 mm. Photochemical cross‐linking was initiated and controlled via illumination by blue LED at a current of 0.03 Amps. To prevent evaporation, during this process low viscosity silicone oil (≈5 ct) was placed around the geometry. The silicone oil should present no schematic error on rheometric data as this is below the rheometer's torque range. Time sweep gelation measurements were conducted at a frequency and shear strain of 1 Hz and 0.5%, respectively.

### Rheometry Analysis

The rheological gelation curves were fitted in accordance with Equation (7),

(7)

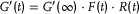

where G’(∞) is the plateau value of G’ at *t* = ∞, F(t) is the formation factor which models the initial hydrogel formation through photochemical cross‐linking, and R(t) is the relaxation factor which models the post‐photochemical cross‐linking relaxation observed in folded protein hydrogels at later times. The formation factor, F(t), is the sum of two sigmoid functions, defined as

(8) $$F\;\left(t\right)=\left(\frac{\alpha }{1+e^{-c^{\alpha }\left(t-t_{0}^{\alpha }\right)}}+\frac{\beta }{1+e^{-c^{\beta }\left(t-t_{0}^{\beta }\right)}}\right)$$

where α + β = 1; c^α^and c^β^ are related to the width of the sigmoid functions, i.e., how steeply the sigmoid grows with time for the α and β formation process, respectively; and finally t_0_^α^ and t_0_^β^ are the sigmoid centers of the α and β formation process, respectively.

The relaxation factor, R(t), is defined as:

(9) $$R\;\left(t\right)=\left(1+B_{1}e^{-\frac{t}{\tau_{1}}}\right)$$

here, τ_1_ is the characteristic relaxation timescale due to network relaxation.

### Lattice‐Based Kinetic Simulations

The coarse‐grained kinetic lattice‐based model simulated the diffusion and aggregation of clusters of cubic units, where each such cubic unit represented one BSA protein monomer. During initialization these units were added sequentially to a periodic simulation cell at random locations, with the constraint that no two monomers were allowed to overlap. Monomer addition continued until the target volume fraction was reached, generating a uniform distribution of “clusters,” each initially consisting of one monomer. During a simulation run, all clusters were randomly displaced to adjacent lattice sites mimicking diffusion, and reacted (cross‐linked, i.e., merging two smaller clusters into a larger cluster) when the surfaces of two different clusters became adjacent, with a probability R per unit time. In this manner, increasingly extended clusters were formed, through gelation, to a final network in which every monomeric unit belonged to a single cluster. The lattice spacing corresponds to the protein diameter, and one unit of simulation time corresponds to the time it takes one protein to diffuse one lattice space. For this work, simulations were run at monomeric volume fractions of 8% and at varying reaction probabilities from 0.2% to 100%, with 10 repeats for each reaction rate. Further details can be found in previous work by Cook et al.^[^ ^49^ ^]^

## Conflict of Interest

The authors declare no conflict of interest.

## Supporting information



Supporting Information

## Data Availability

The data that support the findings of this study are openly available at [https://doi.org/10.5518/1587].
