# Supplementary material for: Capturing Dynamic Assembly of Nanoscale Proteins During Network Formation
Source: Small. 2024 Nov 12;21(1):2407090. doi: 10.1002/smll.202407090 (PMC11707584; doi:10.1002/smll.202407090)
Supplement: Supplementary file 1 — Supporting Information [file SMLL-21-2407090-s001.docx]

Supplementary Materials for

**Capturing Dynamic Assembly of Nanoscale Proteins During Network Formation**

Matt D G Hughes^1^, Kalila R Cook^1^, Sophie Cussons^2,3^, Ahmad Boroumand^1^, Arwen I I Tyler^4^, David Head^5^, David J Brockwell^2,3^, and Lorna Dougan*^1,2^

1 School of Physics and Astronomy, Faculty of Engineering and Physical Sciences, University of Leeds, UK

2 Astbury Centre for Structural Molecular Biology, University of Leeds, UK

3 School of Molecular and Cellular Biology, Faculty of Biological Sciences, University of Leeds, UK

4 School of Food Science and Nutrition, Faculty of Environment, University of Leeds, UK

5 School of Computing, Faculty of Engineering and Physical Sciences, University of Leeds, UK

Corresponding Author: Lorna Dougan, L.Dougan@leeds.ac.uk

**This PDF file includes:**

Supplementary Text showing the derivation of equations to extract k_max­_ and lag time.

Figs. S1 to S4

Supplementary Text

Supplementary Derivations Derivation of sigmoidal lag time (Eqn. 2) and max formation rate (Eqn. 3).

Here we consider the generic form of the sigmoid function, x(t)

| $x\left( t \right)=\frac{\Delta x}{1+e^{-c(t-t_{0})}}+x(0)$ | (S1) |
| --- | --- |

Where c and t_0_ are arbitrary constants and Δx is equal to x(∞) minus x(0). Taking the first derivative of x(t) as

$$\frac{dx}{dt}=\Delta x\cdot c\cdot e^{-c\left( t-t_{0} \right)}\cdot\left( 1+e^{-c\left( t-t_{0} \right)} \right)^{-2}$$

Setting t equal to t_0_ the maximum gradient of the sigmoid, k_max_, (Eqn. 3) can be found

$$\frac{dx\left( t_{0} \right)}{dt}=K_{max}=\frac{\Delta x\cdot c}{4}$$

To determine the lag time, t_gel_, of the sigmoid we linearly extrapolate k_max_ to produce equation S2.

| $y\left( t \right)=K_{max}\cdot t+b$ | (S2) |
| --- | --- |

is equal to x(0), where $b=\frac{1}{4}(2\Delta x+4x\left( 0 \right)-\Delta x\cdot c\cdot t_{0})$ (found by substituting t = t_0_ into equation S1). By definition at the lag time, t = t_gel_, equation S2 is equal to x(0), substituting this into S2 and rearranging we find that

$$t_{gel}=t_{0}-\frac{2}{c}$$

Supplementary Figures

| **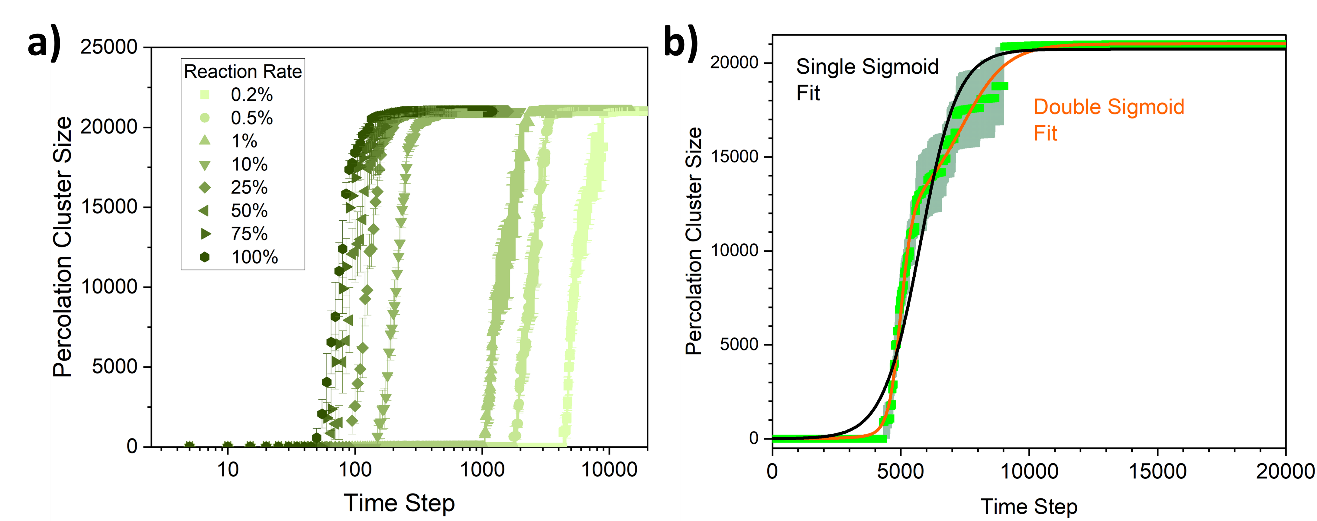** |
| --- |
| **Figure S1: a)** Change in number of monomers in the percolating cluster over time for 8% monomer volume fraction systems ranging from 0.2 to 100% monomer–monomer reaction probabilities. **b)** Exemplar simulated cluster growth curved fitted with a single sigmoid growth model (solid black line) and a double sigmoid growth model (solid orange fit). |

| **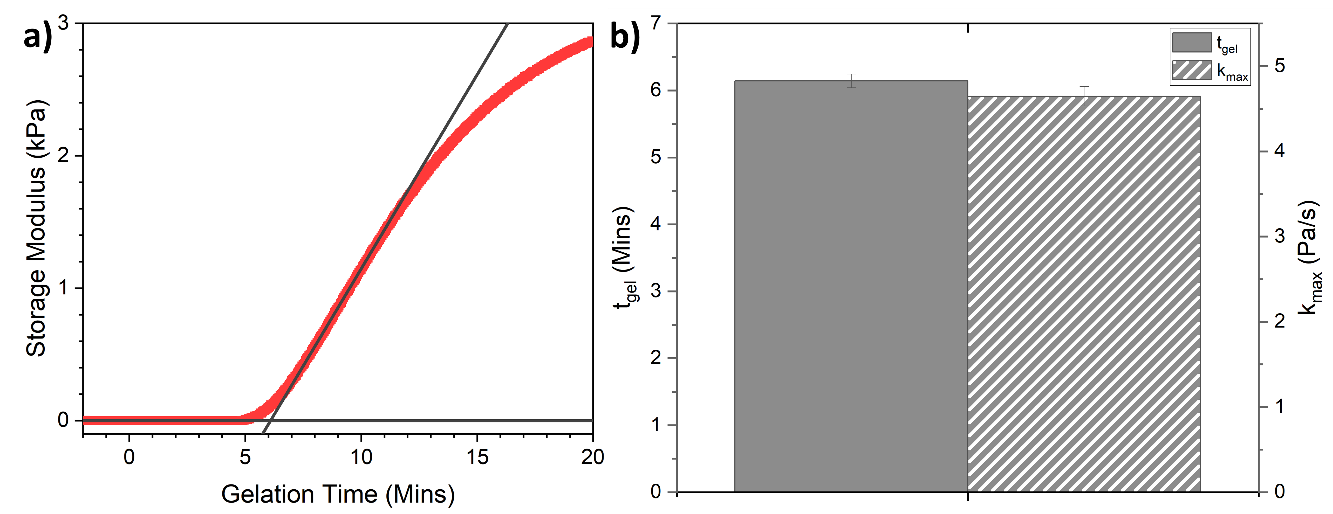** |
| --- |
| **Figure S2: a)** Graph for the conventional extraction of the lag time and maximal gelation rate from rheological gelation curves. Where the red points are the gelation profile of BSA hydrogels (final concentrations: 100 mg/mL BSA, 50 mM NaPS, 100 μM Ru(BiPy)_3_), and the black lines show the fitted linear functions. **b)** The conventionally extracted (as depicted in panel **a)**) lag time (left, solid) and maximum gelation rate (right, striped). |

| 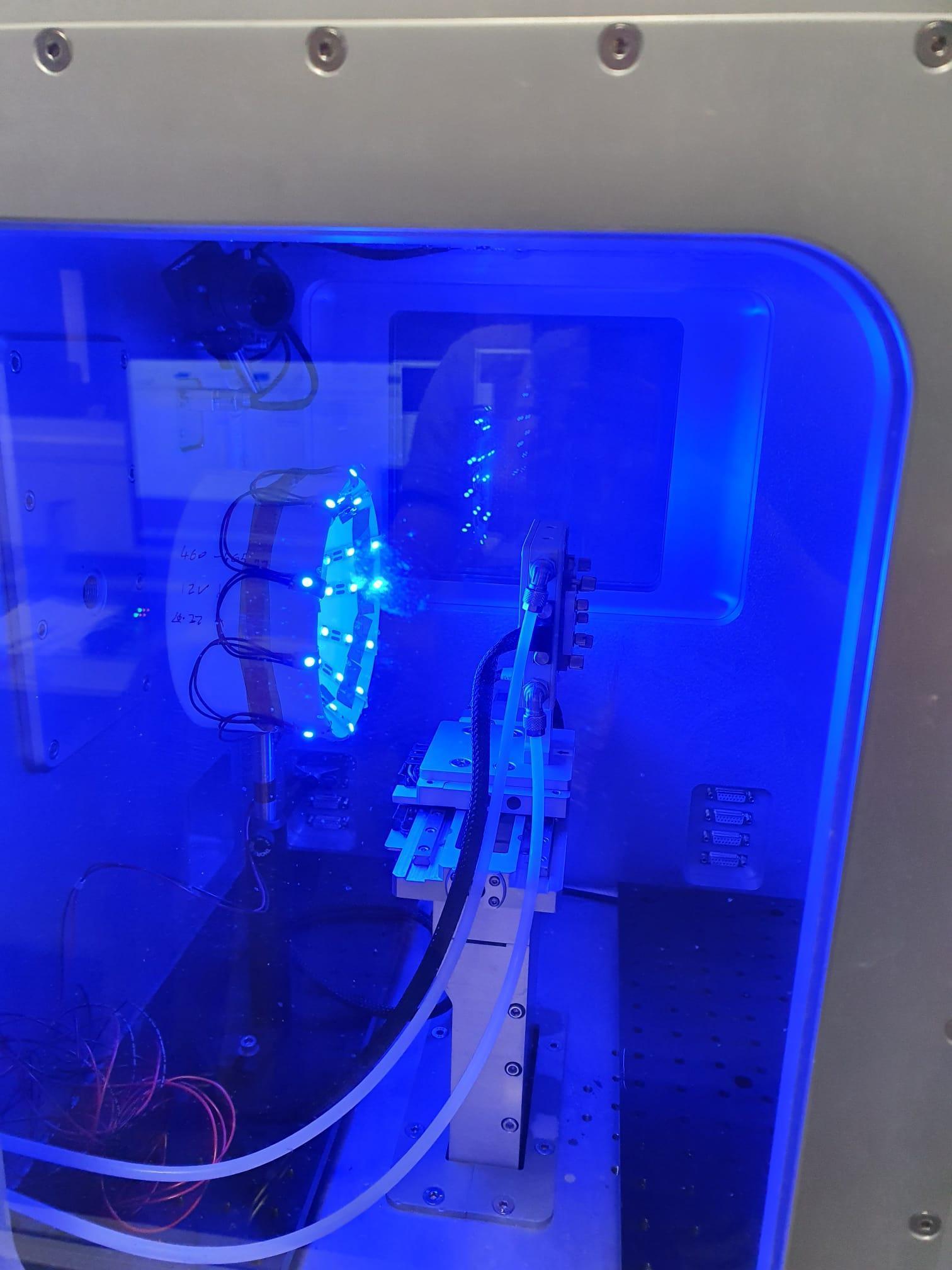 |
| --- |
| **Figure S3:** The bespoke blue LED lighting rig shown *in situ* in the SAXS vacuum sample chamber with Xenocs Peltier Capillary Stage, consisting of a 3D printed cone with 12V SMD3528 LED strips attached for a total of 99 LEDs. |

| 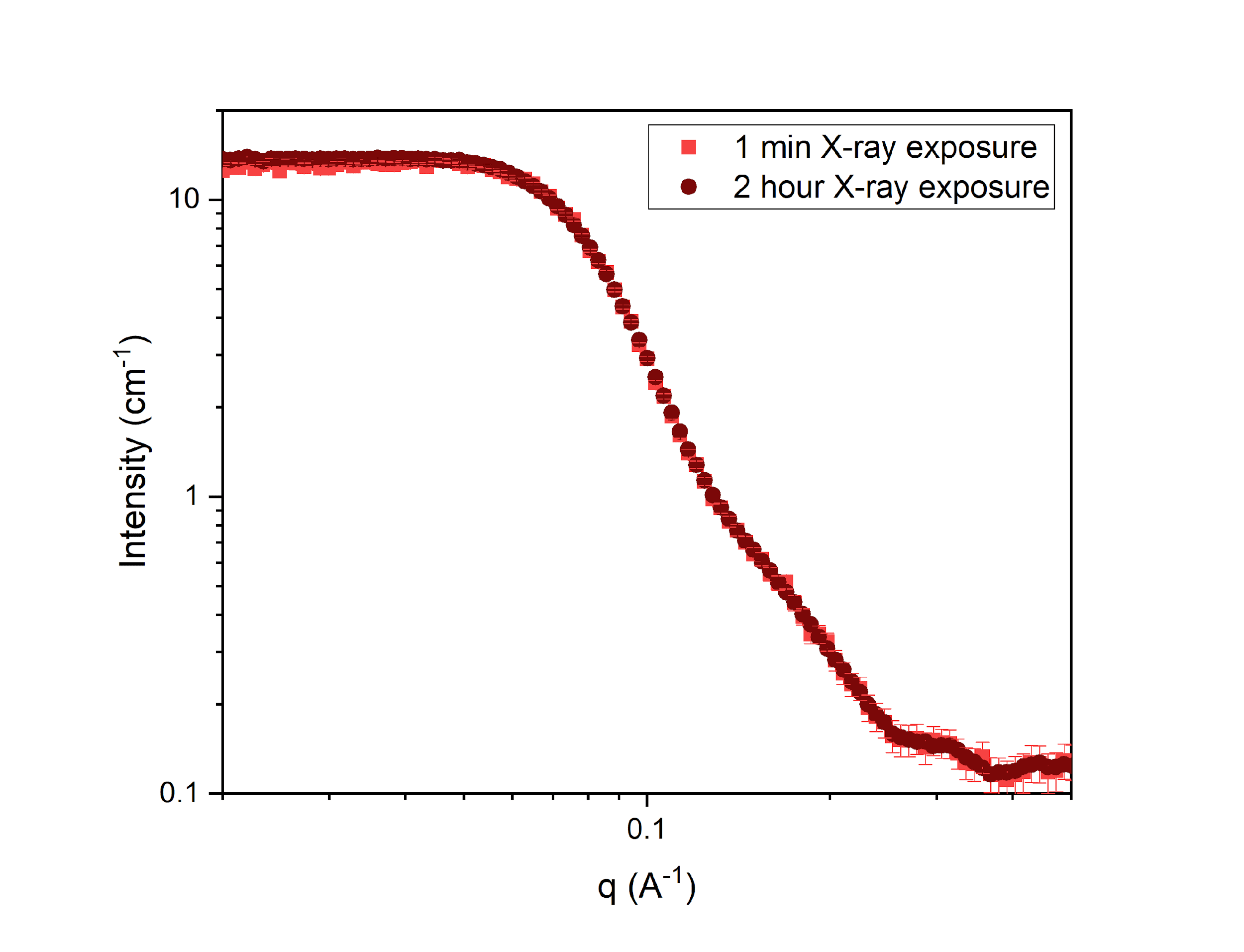 |
| --- |
| **Figure S4: The exposure to x-rays during the experiment are not sufficient to cause radiation damage or significant crosslinking which leads to structural changes.** SAXS curves of BSA pre-gel solutions (final concentrations: 100 mg/mL BSA, 50 mM NaPS, 100 μM Ru(BiPy)_3_) exposed to 1min of X-rays (light red) and 2 hours X-rays (dark red). |
